# Supplementary material for: Current and future state of evaluation of large language models for medical summarization tasks
Source: Npj Health Syst. 2025 Feb 3;2:6. doi: 10.1038/s44401-024-00011-2 (PMC11928168; doi:10.1038/s44401-024-00011-2)
Supplement: Supplementary file 1 — Supplementary Information [file 44401_2024_11_MOESM1_ESM.pdf]

Supplementary Table 1

Database	Query
ACL Anthology	["Electronic Health Records", "health record" , "medical record", "clinical narrative" , "clinical domain" , "medical domain" , "clinical note" , "clinical record" , "clinical text" , "medical text"] AND ["Natural Language Processing", "natural language", "language processing", "NLP", "generative", "generation" , "summarization", "summarisation", "Large Language Model", "LLM"] AND ["evaluation", "human evaluation", "automatic evaluation"]
Embase (Scopus)	((TITLE-ABS-KEY ("Electronic Health Records" OR "health record" OR "medical record" OR "clinical narrative" OR "clinical domain" OR "medical domain" OR "clinical note" OR "clinical record" OR "clinical text" OR "medical text") AND TITLE-ABS-KEY ("Natural Language Processing" OR "natural language" OR "language processing" OR "NLP" OR "generative" OR "generation" OR "summarization" OR "summarisation" OR "Large Language Model" OR "LLM") AND TITLE-ABS-KEY ("evaluation" OR "human evaluation" OR "automatic evaluation"))) AND (INDEX (embase))
PubMed / MEDLINE	(("Electronic Health Records"[MeSH Terms] OR "health record" OR "medical record" OR "clinical narrative" OR "clinical domain" OR "medical domain" OR "clinical note" OR "clinical record" OR "clinical text" OR "medical text") AND ("Natural Language Processing"[MeSH Terms] OR "natural language" OR "language processing" OR "NLP" OR "generative" OR "generation" OR "summarization" OR "summarisation" OR "Large Language Model" OR "LLM") AND ("evaluation" OR "human evaluation" OR "automatic evaluation"))

Table 1: **Search Queries by Database for Searches Completed from April 20, 2023 - August 3, 2023**

Supplementary Table 2

Database	Query
ACL Anthology	["Human Alignment", "Identity Preference Optimization", "Direct Preference Optimization", "Kahneman-Tversky Optimization", "Proximal Policy Optimization", "Reinforcement Learning Human Feedback", "human-centered loss functions"] AND ["Natural Language Processing", "natural language", "language processing", "NLP", "generative", "generation" , "summarization", "summarisation", "Large Language Model", "LLM"]
Embase (Scopus)	((TITLE-ABS-KEY ("Natural Language Processing" OR "natural language" OR "language processing" OR "NLP" OR "generative" OR "generation" OR "summarization" OR "summarisation" OR "Large Language Model" OR "LLM") AND TITLE-ABS-KEY ("Human Alignment" OR "Identity Preference Optimization" OR "Direct Preference Optimization" OR "Kahneman-Tversky Optimization" OR "Proximal Policy Optimization" OR "Reinforcement Learning Human Feedback" OR "human-centered loss function"))) AND (INDEX (embase))
PubMed / MEDLINE	(("Natural Language Processing"[MeSH Terms] OR "natural language" OR "language processing" OR "NLP" OR "generative" OR "generation" OR "summarization" OR "summarisation" OR "Large Language Model" OR "LLM") AND ("Human Alignment" OR "IPO" OR "DPO" OR "KTO" OR "PPO" OR "RLHF" OR "Identity Preference Optimization" OR "Direct Preference Optimization" OR "Kahneman-Tversky Optimization" OR "Proximal Policy Optimization" OR "Reinforcement Learning Human Feedback" OR "human-centered loss function" OR "HALO"))
Web of Science	TS=("Natural Language Processing" OR "natural language" OR "language processing" OR "NLP" OR "Large Language Model" OR "LLM" OR "Language Model") AND TS=("Human Alignment" OR "Identity Preference Optimization" OR "Direct Preference Optimization" OR "Kahneman-Tversky Optimization" OR "Proximal Policy Optimization" OR "Reinforcement Learning Human Feedback" OR "human-centered loss function")
IEEE Xplore	(("Natural Language Processing" OR "natural language" OR "language processing" OR "NLP" OR "Large Language Model" OR "LLM") AND ("Human Alignment" OR "Identity Preference Optimization" OR "Direct Preference Optimization" OR "Kahneman-Tversky Optimization" OR "Proximal Policy Optimization" OR "Reinforcement Learning Human Feedback" OR "human-centered loss function"))
ACM Digital Library	[Abstract: "natural language processing"] OR [Abstract: "natural language"] OR [Abstract: "language processing"] OR [Abstract: "nlp"] OR [Abstract: "generative"] OR [Abstract: "generation"] OR [Abstract: "summarization"] OR [Abstract: "summarisation"] OR [Abstract: "large language model"] OR [Abstract: "llm"]] AND [[Abstract: "human alignment"] OR [Abstract: "identity preference optimization"] OR [Abstract: "direct preference optimization"] OR [Abstract: "kahnemann-tversky optimization"] OR [Abstract: "proximal policy optimization"] OR [Abstract: "reinforcement learning human feedback"] OR [Abstract: "human-centered loss function"]

Table 2: **Search Queries by Database for Searches Completed April 16, 2024 - June 6, 2024**
